# Diagnosis and Treatment of Myeloproliferative Neoplasms With PCM1-JAK2 Rearrangement: Case Report and Literature Review

**DOI:** 10.3389/fonc.2021.753842

**Published:** 2021-10-11

**Authors:** Yingxin Sun, Yifeng Cai, Jia Chen, Jiannong Cen, Mingqing Zhu, Jinlan Pan, Depei Wu, Aining Sun, Suning Chen

**Affiliations:** ^1^ Department of Hematology, First Affiliated Hospital of Soochow University, National Clinical Research Center for Hematologic Diseases, Jiangsu Institute of Hematology, Soochow University, Suzhou, China; ^2^ Department of Thrombosis and Hemostasis, Key Laboratory of Thrombosis and Hemostasis of Ministry of Health, Suzhou, China; ^3^ Department of Hematology, The Affiliated Hospital of Nantong University, Nantong University, Nantong, China

**Keywords:** myeloproliferative neoplasms, PCM1-JAK2, ruxolitinib, pegylated interferon, case report

## Abstract

Myeloproliferative neoplasm (MPN) with PCM1-JAK2 rearrangement is a rare disease with poor prognosis and lacks uniform treatment guidelines. Several studies confirmed the efficacy of ruxolitinib in hematological malignancies with PCM1-JAK2 fusion, but the efficacy is variable. Here, we report two patients diagnosed with MPN with PCM1-JAK2 fusion who were treated with ruxolitinib-based regimen, including the first case of ruxolitinib combined with pegylated interferon (Peg-IFN), and we conduct a literature review. We found that ruxolitinib combined with Peg-IFN is an effective treatment option in the case of poor efficacy of ruxolitinib monotherapy.

## Introduction

JAK2 components play an important role in hematopoiesis, cell proliferation, and differentiation. Abnormal activation of JAK pathway by gene mutations or rearrangements is common in Philadelphia-negative myeloproliferative neoplasm (MPN). About 75% of typical Philadelphia-negative MPN, including essential thrombocytosis (ET), polycythemia vera (PV), and primary myelofibrosis (PMF), carry a specific V617F somatic mutation in JAK2 gene (1, 2). In contrast, chromosomal translocations involving JAK2 gene are rare and have been reported in various hematological malignancies. Among them, the PCM1-JAK2 fusion gene derived from t(8;9)(p22;p24) is the most frequent (3–7). MPN with PCM1-JAK2 rearrangement is accompanied by varying degrees of eosinophilia, lymphadenopathy/hepatosplenomegaly, and myelofibrosis (8). Based on share characteristics, myeloid/lymphoid neoplasms (MLNs) with PCM1-JAK2 rearrangement has been added as a provisional entity in the 2016 World Health Organization (WHO) classification of myeloid neoplasms and acute leukemia (9).

MLN with PCM1-JAK2 rearrangement is a rare disease with poor prognosis and lacks unified treatment guidelines. Up to now, allogeneic hematopoietic stem cell transplantation (allo-HSCT) is the only way that can cure the disease. Ruxolitinib, a JAK2 inhibitor, has been approved by the United States Food and Drug Administration for the treatment of intermediate- and high-risk PMF according to the findings of two randomized controlled trials (10, 11). However, ruxolitinib is not yet approved for MPN with PCM1-JAK2 rearrangement, although it has been shown to be effective. Here, we report two patients with MPN with PCM1-JAK2 fusion who received ruxolitinib-based regimen, one of which is the first report of ruxolitinib combined with pegylated interferon (Peg-IFN) at home and abroad.

## Case Presentation

### Case 1

In October 2020, a 42-year-old man was referred to our department because of leukocytosis (leukocyte 48.4 × 10^9^/L) with eosinophilia (eosinophils 3.97 × 10^9^/L), mononucleosis (monocyte 1.9 × 10^9^/L), and anemia (hemoglobin 106 g/L). The platelet count is normal (159 × 10^9^/L). The morphological test of peripheral blood showed that eosinophils accounted for 9%, monocytes accounted for 9%, and blasts accounted for 1%. Ultrasound showed that the spleen is slightly larger (length 13.2 cm) without lymphadenopathy and hepatomegaly. The bone marrow analyses showed hypercellular morphology (myeloblast 2%, monoblast 2%, promonocyte 9%, and eosinophilia) with dyshematopoiesis in granule and erythroid linages, but no evidence of myelofibrosis. Bone marrow immunohistochemistry: myeloperoxidase (3+), glycophorin A (2+), CD3 (−), CD20 (−), CD38 (−), and CD34 (−). Chromosomal analysis showed a karyotype of 46,XY,t(8;9). Targeted next-generation sequencing (NGS) was negative. RNA sequencing revealed that exon 36 of PCM1 was fused to exon 8 of JAK2. In conclusion, the patient was diagnosed with MLN with eosinophilia (MLN-Eo) and PCM1-JAK2 rearrangement according to the 2016 WHO criteria (12).

**Figure 1:**
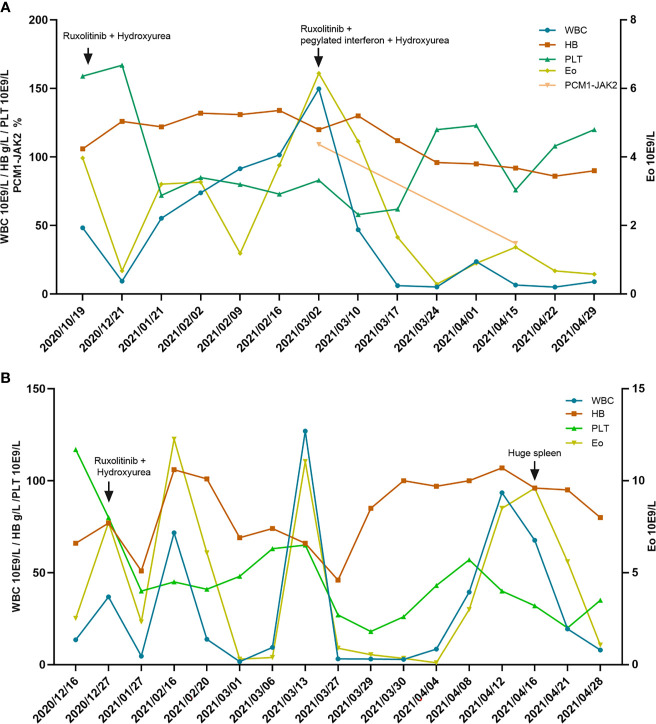
**(A)** The blood cell and PCM1-JAK2 fusion transcript changes in patient 1 over the course of the disease. **(B)** The blood cell changes in patient 2 over the course of the disease. WBC, white blood cells; HB, hemoglobin; PLT, platelet; Eo, eosinophils.

Induction treatment for the patient was hydroxyurea (HU; 500 mg qd) combined with ruxolitinib (with initiating dose of 5 mg qd and then escalated to 15 mg bid). The patient achieved complete hematologic remission (CHR) in 1 month and then accepted the maintenance therapy with ruxolitinib alone. Subsequently, his leukocytes and eosinophils progressively increased, accompanied by a reduction of platelet counts. Two and a half months after stopping HU, the patient complained of abdominal distension, and abdominal Doppler ultrasound indicated that the spleen was 3.5 cm below the ribs. PCM1-JAK2 quantitative PCR test indicated 109.17% (no data at diagnosis). In addition to ruxolitinib, Peg-IFN (90 μg s.c. qw) and HU (500 mg bid) were administered. The patient tolerated the combined treatment well. Assessment conducted 2 months later showed that leukocytes, eosinophils, and platelets were significantly improved, the spleen size returned to normal, and PCM1-JAK2 fusion transcript decreased to 37.03%. These results suggested that the combination of ruxolitinib and Peg-IFN was safe and effective. Currently, the patient is still receiving combined therapy, and he is planning to undergo haploidentical HSCT (**Figure 1A**).

### Case 2

In December 2020, a 47-year-old man was referred to the hematological department complaining of fatigue, tinnitus, and dizziness for 2 months. Count of blood cells test showed leukocytes of 13.47 × 10^9^/L, eosinophils of 2.52 × 10^9^/L, severe anemia (hemoglobin 66 g/L), and normal platelet counts (117 × 10^9^/L). Peripheral blood smear revealed the presence of immature granulocytes and erythrocytes, with teardrop-like erythrocytes and 14% eosinophils. Physical examination suggested hepatosplenomegaly. The bone marrow morphology was hypercellular and showed granulocytic proliferation with eosinophilic proliferation, reduced erythropoiesis, hyperplasia of megakaryocytes, and grade 0-1 fibrosis according to European Myelofibrosis Network criteria (13). Cytogenetic analysis demonstrated a normal male karyotype. NGS was negative. Fluorescence *in situ* hybridization study demonstrated that BCR-ABL1, BCR-JAK2, ETV6-JAK2, ETV-FLT3, ETV-ABL1, PDGFRA, PDGFRB, and FGFR1 fusion and rearrangement were all negative. The PCM1-JAK2 fusion transcript was identified by reverse transcription polymerase chain reaction (RT-PCR). The break site was located in exon 36 of PCM1 and exon 9 of JAK2. The diagnosis of MLN-Eo with PCM1-JAK2 fusion was established. The patient returned to the local hospital for treatment and was treated with HU (500 mg tid), ruxolitinib (15 mg bid), and red blood cell transfusion after patient informed consent. According to the white blood cell counts, HU was discontinued after 0.5 months. One month later, the patient’s white blood cells (1.66 × 10^9^/L) and eosinophils (0.30 × 10^9^/L) decreased significantly, the hepatosplenomegaly was improved, and the platelet (48 × 10^9^/L) decreased, so the dose of ruxolitinib was reduced. However, both white blood cells and eosinophils were progressively increased since the dose of ruxolitinib was reduced. The final dose of ruxolitinib was maintained at 10 and 15 mg alternately every day. During the treatment of ruxolitinib, there was Grade 3 leukopenia and Grade 4 thrombocytopenia. Later, the patient developed a huge spleen, which was considered to be related to the insufficient dose of ruxolitinib. In short, the patient cannot tolerate the therapeutic dose of ruxolitinib and is preparing for allo-HSCT (**Figure 1B**).

## Discussion

**Figure 2:**
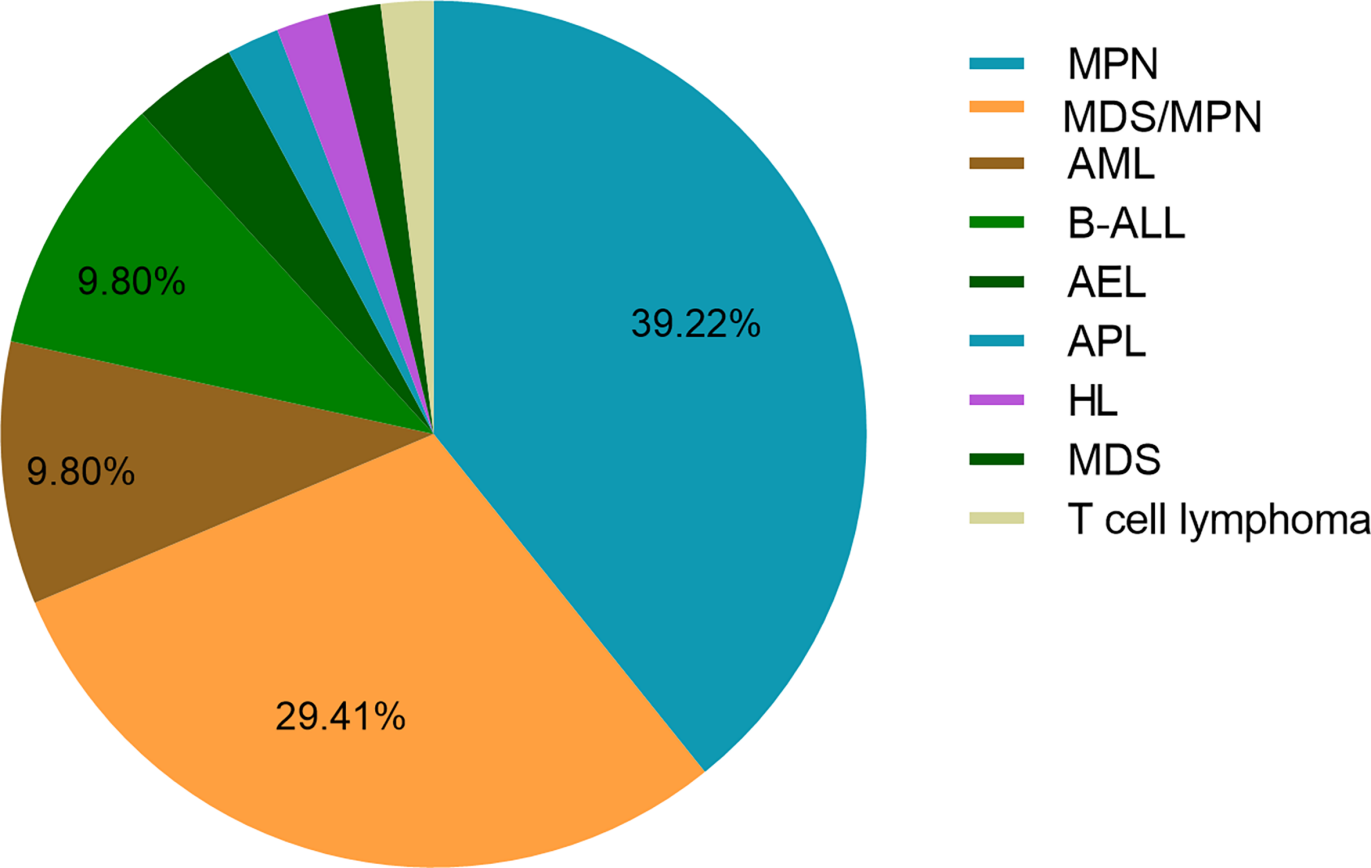
Distribution of PCM1-JAK2 fusion in hematological neoplasms in the literature. MPNs, myeloproliferative neoplasms; MDS/MPN, myelodysplastic syndrome/myeloproliferative neoplasms; AML, acute myeloid leukemia; B-ALL, B-cell acute lymphoblastic leukemia; AEL, acute erythroid leukemia; APL, acute promyelocytic leukemia; HL, Hodgkin lymphoma.

So far, 68 cases of PCM1-JAK2 fusion have been reported in hematological malignancies, most commonly in myelodysplastic syndrome (MDS)/MPN and MPN. The median age is 50 years (12–86 years), the male to female ratio is 3.17:1, 83.61% of patients have varying degrees of eosinophilia, and 50% of patients have myelofibrosis (**Table 1** and **Figure 2**). Thirteen patients were treated with ruxolitinib ± HSCT, of which 11 were myeloid neoplasms and two were lymphatic neoplasms. Twelve were responsive to ruxolitinib, and one was of uncertain efficacy; however, the duration of response varied widely (**Table 2**). The 2021 National Comprehensive Cancer Network (NCCN) guidelines for MLN-Eo with PCM1-JAK2 fusion recommend that clinical trial is the preferred treatment option for patients with chronic phase disease, and patients with chronic phase disease can be treated with tyrosine kinase inhibitor (TKI) monotherapy in the absence of a clinical trial. However, early referral to allo-HSCT should be considered for eligible patients, since TKI therapy alone does not result in durable remissions. We report two male cases diagnosed as MLN-Eo with PCM1-JAK2 fusion. Patient 1 was initially treated with ruxolitinib and HU to obtain instant CHR but later experienced progressive increase of white blood cells and eosinophils, which indicated treatment failure. The effect was regained after the addition of Peg-IFN, and PCM1-JAK2 fusion transcript decreased significantly, suggesting that molecular response was obtained. As far as we know, this is the first report of MLN-Eo and PCM1-JAK2 fusion receiving ruxolitinib combined with Peg-IFN at home and abroad. Unfortunately, limited by short follow-up time, we have not observed patients obtained complete molecular response (CMR). Patient 2 initially responded to treatment with ruxolitinib and HU, but it was discontinued due to intolerant hematological toxicity. Both cases indicated the effectiveness of ruxolitinib in MLN-Eo with PCM1-JAK2 fusion, despite different durations of response and toxicity, and both patients will undergo HSCT.

**Table 1:** Hematological neoplasms with PCM1-JAK2 fusion from literature.

Authors	Time	Journals	Number of cases
Reiter et al. (5)	2005	*Cancer Research*	7
Bousquet et al. (4)	2005	*Oncogene*	2
Murati et al. (3)	2005	*Leukemia*	4
Heiss et al. (14)	2005	*Human Pathology*	1
Adélaïde et al. (6)	2006	*Leukemia*	1
Huang et al. (15)	2008	*International Journal of Hematology*	1
Dargent et al. (16)	2011	*European Journal of Haematology*	1
Prochorec-Sobieszek et al. (17)	2012	*Leukemia & Lymphoma*	1
Lierman et al. (18)	2012	*Blood*	1
Masselli et al. (19)	2013	*British Journal of Haematology*	1
Patterer et al. (20)	2013	*Annals of Hematology*	6
Rumi et al. (21)	2013	*Journal of Clinical Oncology*	1
Saba et al. (22)	2013	*Blood*	1
Schwaab et al. (23)	2015	*Annals of Hematology*	1
Song et al. (24)	2016	*Annals of Laboratory Medicine*	1
Baer et al. (25)	2018	*Haematologica*	7
Lee et al. (26)	2018	*Annals of Laboratory Medicine*	1
Riedlinger et al. (7)	2019	*JCO Precision Oncology*	1
Salehi et al. (27)	2019	*Leukemia & Lymphoma*	1
Tang et al. (8)	2019	*Modern Pathology*	10
Schwaab et al. (28)	2020	*American Journal of Hematology*	8
Wouters et al. (29)	2021	*British Journal of Haematology*	1
Pozdnyakova et al. (30)	2021	*American Journal of Clinical Pathology*	9

**Table 2:** Clinical characteristics of hematological neoplasms patients with PCM1-JAK2 rearrangement treated with ruxolitinib in the literature.

Case no.	Age	Gender	Diagnosis	WBC (×10^9^/L)	Eo (%)	Splenomegaly	Bone marrow	Karyotype	Treatment	Clinical course
A1	72	M	CEL, NOS	49	46.9	NA	Granulocytosis and eosinophilia	t(8;9)(p22;p24)	HU and ruxolitinib	CCyR was obtained after 15 months on ruxolitinib.
B2	50	M	MPN	9.9	6	Yes	Granulopoiesis, left-shifted and eosinophilia	t(8;9)(p22;p24)	Ruxolitinib	Alive 16 months after diagnosis.
C3	31	F	CEL, NOS	21.6	16.2	Yes	Granulopoiesis, eosinophilia, immature erythroid cells, and MF 1	t(8;9)(p22;p24)	Imatinib, HU, and ruxolitinib	No response to imatinib; then complete clinical remission was achieved after ruxolitinib.
D4	51	M	MPN	12	NA	Yes	Granulopoiesis, eosinophilia, dysplastic erythropoiesis, and MF 2	t(8;9)(p22;p24)	Ruxolitinib	CCyR was obtained after 12 months on ruxolitinib, but cytogenetic relapse occurred after 24 months.
E5	40	M	MPN	5.4	16	Yes	Eosinophils, immature erythroid precursors and MF 1–2	t(8;9)(p22;p24)	Ruxolitinib and HSCT	No disease progression during the follow-up period.
F6	76	M	CML-like MPN	28.7	5	No	Eosinophilia, left-shifted, dysplastic, and MF 2	t(8;9)(p22;p24)	Ruxolitinib	Disease progression after 1 month on ruxolitinib.
F7	70	M	aCML	29.8	1	Yes	Eosinophilia, left-shifted, dysplastic and MF 1	t(8;9)(p22;p24)	Ruxolitinib	CHR was obtained after 2 months on ruxolitinib.
F8	49	M	MDS/MPN	25.6	NA	Yes	Eosinophilia, left-shifted, dysplastic and MF 2	t(8;9;9)(p22;p24;p13)	Ruxolitinib and HSCT	CHR was obtained after 2 months on ruxolitinib, and the disease progressed 36 months later, followed by HSCT.
F9	29	M	CML-like MPN	21.7	11	Yes	NA	t(8;9)(p22;p24)	Ruxolitinib and HSCT	CHR was obtained after 2 months on ruxolitinib, followed by HSCT.
F10	50	M	MDS/MPN	12.7	13	Yes	Eosinophilia, left-shifted, dysplastic, and MF 2	t(8;9)(p22;p24)	Ruxolitinib and HSCT	CHR was obtained after 18 months on ruxolitinib, and the disease progressed 8 months later, followed by HSCT.
F11	69	F	AML-M4	10.5	1	No	Blasts 20% and MF 3	t(8;9)(p22;p24),+6,+8,+22	Ruxolitinib and azacitidine	The disease progressed after 2 months on ruxolitinib, CHR was obtained after 3 months on azacitidine, and the disease progressed again after 9 months.
F12	63	M	Pre-B-ALL	55.2	NO	No	Sheets of blasts	t(8;9)(p22;p24)	Ruxolitinib and HSCT	HSCT followed by ruxolitinib; then disease progressed.
G13	77	F	B-ALL	32.6	NO	No	87% blasts	t(8;9)(p22;p24)[1]/46,sl,der(8;9)(q10;q10),inc [5]/46,X,t(X;4)(p1?1;q13)[4]/46,XX[10]	Chemotherapy, blinatumomab, and ruxolitinib	PCM1-JAK2 fusion transcript was 23.28% after chemotherapy combined with blinatumomab, which decreased to 3.22% after the addition of ruxolitinib.

M, man; F, female; WBC, white blood cells; Eo, eosinophils; CEL, NOS, Chronic eosinophilic leukemia, not otherwise specified; CML, chronic myeloid leukemia; aCML, atypical chronic myeloid leukemia; CCyR, complete cytogenetic remission; NA, not available. Case A: (Lierman et al.) (18). Case B: (Patterer et al.) (20). Case C: (Rumi et al.) (21). Case D: (Schwaab et al.) (23). Case E: (Tang et al.) (8). Case F: (Schwaab et al.) (28). Case G: (Wouters et al.) (29).

PCM1-JAK2 rearrangement has been reported in a variety of hematological neoplasms (3–7), which indicates that the PCM1-JAK2 rearrangement lacks lineage specificity. Because of the abnormality of the JAK2 signaling pathway in these diseases, treatment with ruxolitinib may be effective. Previous documents confirmed that ruxolitinib can inhibit the growth of PCM1-JAK2 transformed Ba/F3 mouse cells *in vitro* and the phosphorylation of JAK-STAT5 pathway (18). Lierman (18) and colleagues reported the first case of ruxolitinib in myeloproliferative disease with PCM1-JAK2 rearrangement. A 72-year-old male diagnosed with chronic eosinophilic leukemia (CEL) of PCM1-JAK2 rearrangement received ruxolitinib alone 10–20 mg twice daily, achieved complete cytogenetic response (CCyR) after 15 months, and was recurrence-free after 36 months (31). Subsequently, Rumi et al. (21) reported a second similar case. A 31-year-old woman was diagnosed with CEL with PCM1-JAK2 rearrangement. She was treated with ruxolitinib 15 mg twice, acquired complete clinical remission 1 year later, and attained CCyR 46 months later, with a significant decrease in the fusion transcript (31). These cases indicated that ruxolitinib is valuable for MPN with PCM1-JAK2 rearrangement and could induce long-term remission. Schwaab et al. (23) identically confirmed that ruxolitinib was valuable in myeloid neoplasms with PCM1-JAK2 fusion, but the patient relapsed after 24 months on ruxolitinib. Recently, Schwaab et al. (28) reported a series of nine myeloid malignancy cases treated with ruxolitinib alone as first-line treatment, including eight cases of PCM1-JCK2 rearrangement and one case of BCR-JAK2 rearrangement. With a median time of 4 months (range 2–18 months), five patients achieved CHR. CCyR or CMR was observed in one patient. Their data showed that all the patients did not have long-term beneficial effects with ruxolitinib. In brief, the efficacy of ruxolitinib in MPN with PCM1-JAK2 fusion is inconsistent, with some patients surviving for a long time after ruxolitinib treatment and others relapsing early. There are also differences in the efficacy of ruxolitinib in lymphoid neoplasms with abnormal JAK2 pathway. Recent data (29) reported that an elderly woman diagnosed with B-cell acute lymphoblastic leukemia (B-ALL) with PCM1-JAK2 rearrangement failed to obtain complete cytogenetic and molecular biological response after receiving traditional chemotherapy and immunotherapy. Within 1 year after ruxolitinib 10 mg bid treatment, the PCM1-JAK2 fusion transcript and abnormal metaphase were significantly reduced, but none of them disappeared completely. Another young female patient with relapsed and refractory B-ALL with RNPC3-JAK2 fusion did not respond to chemotherapy combined with immunotherapy and ruxolitinib (32). Mayfield et al. (33) documented that a 17-year-old B-ALL patient with JAK2 F694L mutation persisted with minimal residual disease (MRD) after standard chemotherapy, while the MRD turned out to be negative after the integration of ruxolitinib 20 mg bid for 2 weeks. The research of Ding (34) and his coworkers found that high-dose ruxolitinib combined with multidrug chemotherapy was safe and effective for children with BCR-ABL1-like ALL, and whether the combination therapy is suitable for MPN with PCM1-JAK2 fusion is worth exploring. In summary, there have been considerable evidences that ruxolitinib is effective in hematological malignancies with abnormal JAK2 signaling pathways, although the efficacy is highly heterogeneous. The heterogeneity may be partially attributed to different somatic mutations or blastic crisis of MPN (8).

No somatic mutations were detected in both of our patients. Due to the rarity of hematological neoplasms with PCM1-JAK2 rearrangement, it is difficult to conduct large-scale clinical studies. Thus, there are no large cohort data on the molecular characterization of PCM1-JAK2-rearranged hematologic neoplasms. Baer et al. (25) found that mutation rates were 14% (1/7) for hematologic neoplasms with PCM1-JAK2 rearrangement, and the patient had TET2 somatic mutations. We speculate that somatic mutations of epigenetic regulators may be present in hematologic neoplasms with PCM1-JAK2 fusion, so hypomethylating-agent-based programs may be effective. Dargent et al. (16) described the diagnosis and treatment of a patient with MDS/MPN with PCM1-JAK2 fusion. The initial analysis of bone marrow karyotype was normal, but t(8;9) was detected by fluorescence *in situ* hybridization analysis in peripheral blood. The author suggested that t(8;9)(p22;p24) was not easy to detect in the study of G-banding and was easy to be ignored, especially in poor specimens. Tang (8) and his colleagues also noted that because t(9p24.1;V) only involved small segments of the 9p chromosome, such rearrangements were cryptic and therefore missed by routine chromosome analysis. These explain the reason for the normal karyotype of patient 2.

## Conclusion

MLN-Eo with PCM1-JAK2 rearrangement is rare and has a poor prognosis. Existing data have shown that ruxolitinib is effective for the disease, but allo-HSCT is still the only way to cure the disease. Ruxolitinib can be used as a bridging treatment before allo-HSCT. Here, we reported the efficacy and safety of ruxolitinib in MPN with PCM1-JAK2 rearrangement. For patients with low efficacy of ruxolitinib monotherapy or rapid disease progression, the treatment options to obtain cytogenetics or molecular response prior to HSCT warrant further study. Ruxolitinib combined with Peg-IFN could be one of the candidates.

## Data Availability Statement

The original contributions presented in the study are included in the article/supplementary material. Further inquiries can be directed to the corresponding author.

## Ethics Statement

The studies involving human participants were reviewed and approved by the Research Ethics Committee of the First Affiliated Hospital of Soochow University. The patients/participants provided their written informed consent to participate in this study.

## Author Contributions

YS wrote the manuscript. SC guided the treatment of cases. YC, JC, JNC, MZ, and JP performed the research and analyzed the data. All authors contributed to the article and approved the submitted version.

## Funding

This study was supported by grants from the National Key R&D Program of China (2019YFA0111000), the National Natural Science Foundation of China (81700140, 81873449, 81900130, 81970136, 81970142, 81970142, 82000132, and 82000158), the Natural Science Foundation of the Jiangsu Higher Education Institution of China (18KJA320005), the Natural Science Foundation of Jiangsu Province (BK20190180), priority academic program development of Jiangsu Higher Education Institution, the Innovation Capability Development Project of Jiangsu Province (BM215004), the Translational Research Grant of NCRCH (2020WSB03, 2020WSB11, and 2020WSB13), the Open Project of Jiangsu Biobank of Clinical Resources (SBK202003001 and SBK202003003), Jiangsu Provincial Key Medical Center (YXZXA2016002), and National Science and Technology Major Project (2017ZX09304021).

## Conflict of Interest

The authors declare that the research was conducted in the absence of any commercial or financial relationships that could be construed as a potential conflict of interest.

## Publisher’s Note

All claims expressed in this article are solely those of the authors and do not necessarily represent those of their affiliated organizations, or those of the publisher, the editors and the reviewers. Any product that may be evaluated in this article, or claim that may be made by its manufacturer, is not guaranteed or endorsed by the publisher.

## References

[1] James C Ugo V Le Couédic J-P Staerk J Delhommeau F Lacout C. A Unique Clonal JAK2 Mutation Leading to Constitutive Signalling Causes Polycythaemia Vera. Nature (2005) 434:1144–8. doi: 10.1038/nature03546 15793561

[2] Kralovics R Passamonti F Buser AS Teo S Tiedt R Passweg JR. A Gain-of-Function Mutation of JAK2 in Myeloproliferative Disorders. New Engl J Med (2005) 352:1779–90. doi: 10.1056/NEJMoa051113 15858187

[3] Murati A Gelsi-Boyer V Adélaïde J Perot C Talmant P Giraudier S. PCM1-JAK2 Fusion in Myeloproliferative Disorders and Acute Erythroid Leukemia With T(8;9) Translocation. Leukemia (2005) 19:1692–6. doi: 10.1038/sj.leu.2403879 16034466

[4] Bousquet M Quelen C De Mas V Duchayne E Roquefeuil B Delsol G. The T(8;9)(P22;P24) Translocation in Atypical Chronic Myeloid Leukaemia Yields a New PCM1-JAK2 Fusion Gene. Oncogene (2005) 24:7248–52. doi: 10.1038/sj.onc.1208850 16091753

[5] Reiter A Walz C Watmore A Schoch C Blau I Schlegelberger B. The T(8;9)(P22;P24) Is a Recurrent Abnormality in Chronic and Acute Leukemia That Fuses PCM1 to JAK2. Cancer Res (2005) 65:2662–7. doi: 10.1158/0008-5472.CAN-04-4263 15805263

[6] Adélaïde J Pérot C Gelsi-Boyer V Pautas C Murati A Copie-Bergman C. A T(8;9) Translocation With PCM1-JAK2 Fusion in a Patient With T-Cell Lymphoma. Leukemia (2006) 20:536–7. doi: 10.1038/sj.leu.2404104 16424865

[7] Riedlinger GM Chojecki A Aviv H Weissmann D Joshi S Murphy SM. Hodgkin Lymphoma and Cutaneous T-Cell Lymphoma Sharing the PCM1-JAK2 Fusion and a Common T-Cell Clone. JCO Precis Oncol (2019) 1–8. doi: 10.1200/PO.19.00082 PMC6785048 31598574

[8] Tang G Sydney Sir Philip JK Weinberg O Tam W Sadigh S Lake JI. Hematopoietic Neoplasms With 9p24/JAK2 Rearrangement: A Multicenter Study. Modern Pathol (2019) 32:490–8. doi: 10.1038/s41379-018-0165-9 30401948

[9] Gerds AT Gotlib J Bose P Deininger MW Dunbar A Elshoury A. Myeloid/Lymphoid Neoplasms With Eosinophilia and TK Fusion Genes, Version 3.2021, NCCN Clinical Practice Guidelines in Oncology. J Natl Compr Cancer Network (2020) 18:1248–69. doi: 10.6004/jnccn.2020.0042 32886902

[10] Harrison C Kiladjian J-J Al-Ali HK Gisslinger H Waltzman R Stalbovskaya V. JAK Inhibition With Ruxolitinib *Versus* Best Available Therapy for Myelofibrosis. N Engl J Med (2012) 366:787–98. doi: 10.1056/NEJMoa1110556 22375970

[11] Verstovsek S Mesa RA Gotlib J Levy RS Gupta V DiPersio JF. A Double-Blind, Placebo-Controlled Trial of Ruxolitinib for Myelofibrosis. N Engl J Med (2012) 366:799–807. doi: 10.1056/NEJMoa1110557 22375971 PMC4822164

[12] Arber DA Orazi A Hasserjian R Thiele J Borowitz MJ Le Beau MM. The 2016 Revision to the World Health Organization Classification of Myeloid Neoplasms and Acute Leukemia. Blood (2016) 127:2391–405. doi: 10.1182/blood-2016-03-643544 27069254

[13] Thiele J Kvasnicka HM Facchetti F Franco V van der Walt J Orazi A. European Consensus on Grading Bone Marrow Fibrosis and Assessment of Cellularity. Haematologica (2005) 90:1128–32. 16079113

[14] Heiss S Erdel M Gunsilius E Nachbaur D Tzankov A. Myelodysplastic/Myeloproliferative Disease With Erythropoietic Hyperplasia (Erythroid Preleukemia) and the Unique Translocation (8;9)(P23;P24): First Description of a Case. Hum Pathol (2005) 36:1148–51. doi: 10.1016/j.humpath.2005.07.020 16226118

[15] Huang K-P Chase AJ Cross NCP Reiter A Li T-Y Wang T-F. Evolutional Change of Karyotype With T(8;9)(P22;P24) and HLA-DR Immunophenotype in Relapsed Acute Myeloid Leukemia. Int J Hematol (2008) 88:197–201. doi: 10.1007/s12185-008-0113-4 18594780

[16] Dargent J-L Mathieux V Vidrequin S Deghorain X Vannuffel P Rack K. Pathology of the Bone Marrow and Spleen in a Case of Myelodysplastic/Myeloproliferative Neoplasm Associated With T(8;9)(P22;P24) Involving PCM1 and JAK2 Genes. Eur J Haematol (2011) 86:87–90. doi: 10.1111/j.1600-0609.2010.01525.x 21070368

[17] Prochorec-Sobieszek M Nasiłowska-Adamska B Borg K Kopeć I Kos-Zakrzewska K Juszczyński P. Chronic Eosinophilic Leukemia With Erythroblastic Proliferation and the Rare Translocation T(8;9)(P22;P24) With PCM1–JAK2 Fusion Gene: A Distinct Clinical, Pathological and Genetic Entity With Potential Treatment Target? Leukemia Lymphoma (2012) 53:1824–7. doi: 10.3109/10428194.2012.661856 22288769

[18] Lierman E Selleslag D Smits S Billiet J Vandenberghe P. Ruxolitinib Inhibits Transforming JAK2 Fusion Proteins *In Vitro* and Induces Complete Cytogenetic Remission in T(8;9)(P22;P24)/PCM1-JAK2–Positive Chronic Eosinophilic Leukemia. Blood (2012) 120:1529–31. doi: 10.1182/blood-2012-06-433821 22899477

[19] Masselli E Mecucci C Gobbi G Carubbi C Pierini V Sammarelli G. Implication of MAPK1/MAPK3 Signalling Pathway in T(8;9)(P22;24)/PCM1-JAK2 Myelodysplastic/Myeloproliferative Neoplasms. Br J Haematol (2013) 162:563–6. doi: 10.1111/bjh.12392 23701125

[20] Patterer V Schnittger S Kern W Haferlach T Haferlach C. Hematologic Malignancies With PCM1-JAK2 Gene Fusion Share Characteristics With Myeloid and Lymphoid Neoplasms With Eosinophilia and Abnormalities of PDGFRA, PDGFRB, and FGFR1. Ann Hematol (2013) 92:759–69. doi: 10.1007/s00277-013-1695-3 23400675

[21] Rumi E Milosevic JD Casetti I Dambruoso I Pietra D Boveri E. Efficacy of Ruxolitinib in Chronic Eosinophilic Leukemia Associated With a PCM1-JAK2 Fusion Gene. J Clin Oncol (2013) 31:e269–71. doi: 10.1200/JCO.2012.46.4370 23630205

[22] Saba N Safah H. A Myeloproliferative Neoplasm With Translocation T(8;9)(P22;P24) Involving JAK2 Gene. Blood (2013) 122:861. doi: 10.1182/blood-2013-03-487348 24079014

[23] Schwaab J Knut M Haferlach C Metzgeroth G Horny H-P Chase A. Limited Duration of Complete Remission on Ruxolitinib in Myeloid Neoplasms With PCM1-JAK2 and BCR-JAK2 Fusion Genes. Ann Hematol (2015) 94:233–8. doi: 10.1007/s00277-014-2221-y 25260694

[24] Song I Lee D Lee J-H Jang S Huh J-R Seo E-J. A T(8;9)(P22;P24)/PCM1-JAK2 Translocation in a Patient With Myeloproliferative Neoplasm and Myeloid Sarcoma: First Report in Korea. Ann Lab Med (2016) 36:79–81. doi: 10.3343/alm.2016.36.1.79 26522767 PMC4697351

[25] Baer C Muehlbacher V Kern W Haferlach C Haferlach T. Molecular Genetic Characterization of Myeloid/Lymphoid Neoplasms Associated With Eosinophilia and Rearrangement of PDGFRA, PDGFRB, FGFR1 or PCM1-JAK2. Haematologica (2018) 103:e348–50. doi: 10.3324/haematol.2017.187302 PMC6068021 29567772

[26] Lee J-M Lee J Han E Kim M Kim Y Han K. PCM1 - JAK2 Fusion in a Patient With Acute Myeloid Leukemia. Ann Lab Med (2018) 38:492–4. doi: 10.3343/alm.2018.38.5.492 PMC5973928 29797824

[27] Salehi S Astle JM Sadigh S Lake J Aikawa V Tang G. Myeloid Neoplasm With Eosinophilia and PCM1-JAK2 Associated With Acute Promyelocytic Leukemia With PML-RARA. Leukemia Lymphoma (2019) 60:2299–303. doi: 10.1080/10428194.2019.1581927 30806106

[28] Schwaab J Naumann N Luebke J Jawhar M Somervaille TCP Williams MS. Response to Tyrosine Kinase Inhibitors in Myeloid Neoplasms Associated With *PCM1* - *JAK2*, *Bcr-Jak2* And *ETV6-ABL1* Fusion Genes. Am J Hematol (2020) 95:824–33. doi: 10.1002/ajh.25825 32279331

[29] Wouters Y Nevejan L Louwagie A Devos H Dewaele B Selleslag D. Efficacy of Ruxolitinib in B-Lymphoblastic Leukaemia With the PCM1 – JAK2 Fusion Gene. Br J Haematol (2021) 192:1–4. doi: 10.1111/bjh.17340 33502001

[30] Pozdnyakova O Orazi A Kelemen K King R Reichard KK Craig FE. Myeloid/Lymphoid Neoplasms Associated With Eosinophilia and Rearrangements of PDGFRA, PDGFRB, or FGFR1 or With PCM1-JAK2. Am J Clin Pathol (2021) 155:160–78. doi: 10.1093/ajcp/aqaa208 33367495

[31] Rumi E Milosevic JD Selleslag D Casetti I Lierman E Pietra D. Efficacy of Ruxolitinib in Myeloid Neoplasms With PCM1-JAK2 Fusion Gene. Ann Hematol (2015) 94:1927–8. doi: 10.1007/s00277-015-2451-7 26202607

[32] Chen X Wang F Zhang Y Ma X Liu M Cao P. Identification of RNPC3 as a Novel JAK2 Fusion Partner Gene in B-Acute Lymphoblastic Leukemia Refractory to Combination Therapy Including Ruxolitinib. Mol Genet Genomic Med (2020) 8:1–8. doi: 10.1002/mgg3.1110 PMC7057088 31885183

[33] Mayfield JR Czuchlewski DR Gale JM Matlawska-Wasowska K Vasef MA Nickl C. Integration of Ruxolitinib Into Dose-Intensified Therapy Targeted Against a Novel JAK2 F694L Mutation in B-Precursor Acute Lymphoblastic Leukemia. Pediatr Blood Cancer (2017) 64:e26328. doi: 10.1002/pbc.26328 PMC5366086 27860260

[34] Ding YY Stern JW Jubelirer TF Wertheim GB Lin F Chang F. Clinical Efficacy of Ruxolitinib and Chemotherapy in a Child With Philadelphia Chromosome-Like Acute Lymphoblastic Leukemia With GOLGA5-JAK2 Fusion and Induction Failure. Haematologica (2018) 103:e427–31. doi: 10.3324/haematol.2018.192088 PMC6119161 29773603

